# *Mycobacterium triplex* Pulmonary Disease in Immunocompetent Host

**DOI:** 10.3201/eid1010.040217

**Published:** 2004-10

**Authors:** Claudio Piersimoni, Piergiorgio Zitti, Gianna Mazzarelli, Alessandro Mariottini, Domenico Nista, Diego Zallocco

**Affiliations:** * United Hospitals, Ancona, Italy;; † Careggi Hospital, Florence, Italy

**Keywords:** Mycobacterium triplex, pulmonary infection, immunocompetent host, dispatch

## Abstract

*Mycobacterium triplex*, a recently described, potentially pathogenic species, caused disease primarily in immunocompromised patients. We report a case of pulmonary infection due to this mycobacterium in an immunocompetent patient and review the characteristics of two other cases. In our experience, *Mycobacterium triplex* pulmonary infection is unresponsive to antimycobacterial chemotherapy.

Nontuberculous mycobacteria (NTM) are ubiquitous organisms, commonly isolated from environmental and animal sources (*1*), whose pathogenicity may vary according to the host's immune status. Although exposure to NTM frequently causes no symptoms, clinical manifestations may range from hypersensitivity reactions (*2*) to destructive, even fatal, lung disease. In the case of lung disease caused by NTM, clinical and radiologic features are sometimes indistinguishable from those seen in lung disease caused by *Mycobacterium tuberculosis* complex (MTB). The clinical importance of NTM is often difficult to determine, especially in patients with chronic, preexisting lung disease; criteria for diagnosing disease caused by NTM, most recently updated by the American Thoracic Society in 1997 (*3*), need to be properly fulfilled.

*M. triplex* was first described in 1996 (*4*). Investigators reported a group of slowly growing, nonpigmented mycobacteria, resembling *M. simiae* or *M. avium* complex (MAC) in biochemical tests, which did not react with the commercial probe designed for MAC. The primary characterization of this new organism relied on conventional biochemical tests and analysis of mycolic acids with high-performance liquid chromatography (HPLC), but conclusive evidence was based on sequencing the 16S rRNA hypervariable region. In HPLC analysis, *M. triplex* produces a triple-clustered mycolic acid profile closely related to those of *M. simiae*, *M. genavense*, and the recently described *M. sherrisii* (*5*), but practically indistinguishable from that of *M. lentiflavum*, a novel mycobacterium characterized by Springer et al. (*6*). Phylogenetic studies showed that *M. triplex* and *M. lentiflavum* are closely related to *M. simiae* and *M. genavense*.

*M. triplex* has been reported to cause episodic infection in AIDS patients or in those with other immunocompromising diseases (*7**–**9*). We present the case of an apparently immunocompetent patient with pulmonary disease caused by this mycobacterium and review the characteristics of two similar cases.

## The Case

On January 2002, a 54-year-old, HIV-negative, white woman was referred to the outpatient pulmonary service because of persistent cough lasting >2 years and fatigue. She lived in an urban area, had smoked cigarettes (40 packs/year) for several years, but did not report any history of alcoholism or use of immunosuppressive drugs. Results of physical examination and routine laboratory tests, including a standard tuberculin skin test, were unremarkable. A chest x-ray showed considerable fibrotic interstitial changes associated with patchy parenchymal shadowing in both lower fields and multiple thin-walled cavitary lesions (0.7–3.5 cm) in both upper lobes. A computed tomographic (CT) scan of the chest confirmed the above lesions, including multifocal bronchiectases in the right middle lobe and multiple small nodules in the lower lobes. Smears for acid-fast bacilli (AFB) were positive on two bronchial washing samples. These specimens produced negative results when tested by a commercial strand displacement amplification assay (Becton Dickinson Biosciences, Sparks, MD) specific for MTB. Chemotherapy with isoniazid, rifampin, and ethambutol was started. Cultures produced a slow-growing, nonpigmented mycobacterium that was identified in June 2002 as *M. lentiflavum*, according to the HPLC pattern of mycolic acids. One more bronchial specimen collected in mid-June was AFB smear-positive and yielded the same organism as was detected in January. Isolates were considered clinically relevant, and chemotherapy was changed to ethambutol and clarithromycin.

In December 2002, after 6 months of treatment, a CT scan did not show reduction of pulmonary lesions, although the general condition of the patient had slightly improved. Smears from one out of four additional bronchial washing samples were positive for AFB, and all specimens yielded the same mycobacterium previously detected. A more accurate evaluation of clinical isolates showed that their phenotypic pattern was different from that of *M. lentiflavum* by results of some biochemical tests and the absence of pigmentation. Further gene sequencing study of the 16S rDNA showed that our isolates exhibited 100% homology with the reference strain of *M. triplex* (ATCC 70071). In March 2003, although chemotherapy was well tolerated, microbiologic tests of two bronchoalveolar lavage (BAL) specimens continued to be AFB smear- and culture-positive. Consequently, chemotherapy was changed to include levofloxacin, ethambutol, and clarithromycin. In October 2003, chest x-ray examination and a CT scan showed a slight reduction in the size of lung cavities. The patient continues to receive medication, and the radiologic picture has not improved.

## Microbiologic Aspects

After standard N-acetyl-L-cysteine-sodium hydroxide decontamination (*10*), bronchoaspirate washing and BAL specimens were stained with routine Ziehl-Neelsen and cultured by a combination of the radiometric Bactec system (Becton Dickinson Biosciences) and Löwenstein-Jensen medium. Recovered strains were tested with a commercial multiplex line probe assay (Inno-LiPA Mycobacteria v2, Innogenetics, Ghent, Belgium) specific for MAC and 16 other different mycobacterial species (*11*). Further identification studies were performed by mycolic acid HPLC analysis (*12*) and 16S rDNA sequencing (*13*). Drug susceptibility pattern was determined in liquid medium by using the radiometric macrodilution method developed for MAC (*14*).

**Figure 1:**
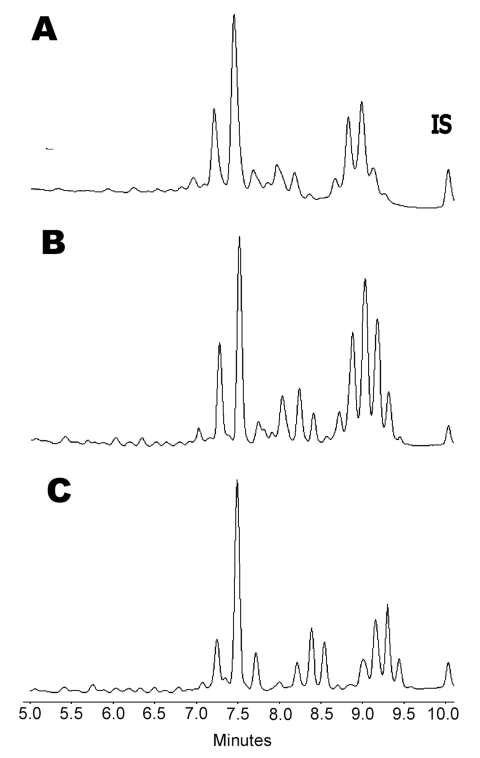
Comparison of high-performance liquid chromatography phenotypes of A) *Mycobacterium triplex*, B) *M. lentiflavum*, and C) *M. simiae*. IS; internal standard.

**Figure 2:**
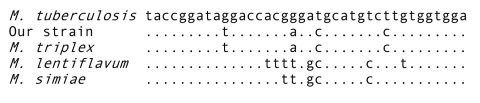
Sequence alignment of the hypervariable region A within the 16s RNA gene of the studied isolate and related species. *Mycobacterium tuberculosis* was used as the reference sequence. Nucleotides different from those of *M. tuberculosis* are indicated; dots indicate identity.

*M. triplex* strains were repeatedly isolated on both liquid and solid media (*17**,**18*). An extended panel of biochemical and cultural tests was used for conventional identification (*10*) (Table 2). The hybridization test performed with the multiplex line probe assay was negative apart from the genus-specific line probe. Final identification was achieved by 16S rDNA gene sequencing. In fact, while HPLC profile was poorly discriminative between *M. lentiflavum* and *M. triplex* (Figure 1), 16S rDNA sequencing showed 100% homology with the *M. triplex* reference strain, which allowed the attribution of all our strains to this species (Figure 2). MICs (µg/mL) performed on the first isolate were the following: streptomycin 6.0, isoniazid >0.5, rifampin >2.0, ethambutol 7.5, ciprofloxacin 4.0, clarithromycin 8.0, amikacin 8.0, ethionamide 2.5, and rifabutin 0.5. Drug susceptibility testing was completed after 6 days. Tentative interpretations of MIC results, according to NCCLS M24-A (*17*) and data from Heifets (*18*), are reported in Table 1.

**Table 2:** Biochemical characteristics of the described isolate compared to those of *Mycobacterium lentiflavum* and *M. triplex*

Characteristic	Our isolate	*M. lentiflavum* (6)	*M. triplex* (4)
Niacin	–	–	–
Nitrate reduction	+	–	+
Thermostable catalase	+	+	+
Tween 80 hydrolysis (10 days)	–	–	–
Tellurite reduction	–	–	NR
Arylsulfatase (3 days)	–	–	–
Urease	+	–	+
Catalase >45 mm	–	–	+
Photochromogenicity	–	–	–
Scotochromogenicity	–	+	–
Growth at 30°C	+	+	+
Growth at 37°C	+	+	+
Growth at 45°C	–	–	–
McConkey w/o CV	–	–	–
Tolerance to NaCl (5%)	–	–	–
Tolerance to TCH (5 mg/mL)	+	+	+
Growth rate	Slow	Slow	Slow
Colonial morphology	Smooth	Smooth	Smooth

^a^–, negative; +, positive; NR, not reported; CV, crystal violet; TCH, thiophene-2-carboxylic acid hydrazide.

**Table 1:** Clinical and microbiological features of pulmonary infection with *Mycobacterium triplex*^a^

Characteristic	Patient described in reference *15*	Patient described in reference *16*	Our patient
Age/Sex	67/F	54/F	54/F
Symptoms	Hemoptysis	Cough, hemoptysis, fever, fatigue	Cough, fatigue
Findings	Bronchiectases, lung nodules	Lung infiltrates and nodule (0.3 cm)	Bronchiectases, lung nodules, cavitations
Collected samples (no.)	Bronchial aspirate (1), sputum (3)	BAL (2), sputum (4)	Bronchial aspirate (7), BAL (2)
Smear-positive	None	None	6
Culture-positive	3	3	9
Mean no. CFU/mL (range)	NR	NR	693 (144–2,772)
In vitro testing			
S	RMP, SM, CLA	NR	CLA
I	CIP	NR	AN, CIP, EMB, ETH, RBT, SM
R	EMB, INH, PZA	NR	INH, RMP
Therapeutic schedule (mo.)	RMP, CIP, EMB, CLA (18)	RMP, INH, CLA (NR)	INH, RMP, EMB (6); EMB, CLA (9); LVX, CLA, EMB (9)
Outcome	Healed	NR	Slight improvement

^a^BAL, bronchoalveolar lavage; NR, not reported; S, susceptible; I, moderately susceptible; R, resistant; RMP, rifampin; SM, streptomycin; CLA, clarithromycin; CIP, ciprofloxacin; AN, amikacin; EMB, ethambutol; ETH, ethionamide; RBT, rifabutin; INH, isoniazid; PZA, pyrazinamide; LVX, levofloxacin.

## Conclusions

A search of the literature from 1996 (when *M. triplex* was first described) to January 2004 yielded two reports of pulmonary infections in immunocompetent patients (*15**,**16*). In those reports, clinical and radiographic features of *M. triplex* pulmonary infection did not differ substantially from those of TB and other NTM. Cough, hemoptysis, and fatigue were the primary symptoms, while radiographic studies found pulmonary nodules most commonly, followed by lung infiltrates, multifocal bronchiectasis, and cavitations (Table 1). No patient had underlying diseases when pulmonary infection with *M. triplex* was diagnosed, and tuberculin skin test results were negative or not reported. Although a history of preexisting pulmonary lesions could not be documented for these patients, repeated isolation of mycobacteria from different respiratory samples and the absence of other possible causes of pulmonary disease suggest that *M. triplex* was likely to cause symptomatic infection rather than colonization (*3*).

*M. triplex* strains were isolated on both liquid and solid media in our case, while culture media were not reported in the other two previously published cases. One case reported an extended panel of biochemical and cultural tests for conventional identification that showed the absence of pigment and positive reactions to nitrate reductase, urease, and semiquantitative catalase as the most useful characteristics for tentative identification (*15*). All strains failed to hybridize with the commercially available genetic probe for MAC (Accuprobe, Gen-Probe Inc., San Diego, CA), so all reported cases could be definitively identified by 16S rRNA gene sequencing and similarity search with the BLAST alignment software (www.blast.genome.ad.jp). One strain showed 100% homology with the reference strain, while another showed homology of 99.5% and, therefore, despite its close relationship, was regarded as a variant of *M. triplex* (*15*).

Patients (including our case-patient) were given different treatment regimens with two to four antimicrobial agents. Ethambutol, rifampin, clarithromycin, and ciprofloxacin were mainly used (Table 1). Clinical improvement, as defined by resolution of symptoms and radiographic findings (infiltrates and cavitary lesions), was obtained within 10 months after therapy was initiated in one of three patients. One patient improved when clarithromycin and ciprofloxacin were added to the regimen, while another was reported to have improved after drug therapy was initiated (*16*). Our patient was still sputum smear and culture–positive 2 years after therapy was initiated, despite exhibiting minor evidence of clinical and radiologic improvement.

Our findings show that *M. triplex* infrequently causes pulmonary disease in immunocompetent persons. Treatment with a three- or four-drug combination, including clarithromycin, ciprofloxacin, and ethambutol, was shown to be associated with reduced symptoms and good clinical outcome. Although at present only 16S rDNA sequencing can identify *M. triplex*, presumptive identification can be made when a slow-growing, nonpigmented NTM reduces nitrates, produces urease and semiquantitative catalase, and exhibits a three-clustered HPLC profile.
